# Population Pharmacokinetics Modeling of Vancomycin Among Chinese Infants With Normal and Augmented Renal Function

**DOI:** 10.3389/fped.2021.713588

**Published:** 2021-09-20

**Authors:** De-Yi Li, Ling Li, Gui-Zhou Li, Ya-Hui Hu, Hong-Li Guo, Xia Jing, Feng Chen, Xing Ji, Jing Xu, Hao-Ran Dai

**Affiliations:** ^1^Pharmaceutical Sciences Research Center, Department of Pharmacy, Children's Hospital of Nanjing Medical University, Nanjing, China; ^2^School of Basic Medicine and Clinical Pharmacy, China Pharmaceutical University, Nanjing, China

**Keywords:** vancomycin, pediatrics, population pharmacokinetics, renal function, external validation

## Abstract

There have been good amounts of population pharmacokinetics (PPK) models of vancomycin for Chinese pediatric patients, but none of them had a special focus on modeling infant population with different levels of renal function. Since renal function variability is prominent among infant population and the clearance (CL) of vancomycin is heavily related to renal excretion, it is important to establish precise PPK models based on individual renal function levels. We employed a PPK approach to develop three models of vancomycin in parallel for Chinese pediatric patients with normal renal function [estimated glomerular filtration rate (eGFR) between 30 and 86 ml/min/1.73 m^2^, Model 1], with augmented renal function (eGFR ≥ 86 ml/min/1.73 m^2^, Model 2), or with all levels of renal function (Model 3). Three one-compartment models with first-order elimination kinetics were established. The predictive ability of Model 1 and Model 2 among each certain population is comparable with that of Model 3 with no statistical difference. Our study revealed that among the infant population with augmented renal function, only body weight was included as a covariate, which indicated that for an infant whose eGFR ≥ 86 ml/min/1.73 m^2^, taking blood sample is not compulsory for predicting vancomycin blood concentration, which avoids unnecessary injury to vulnerable infants.

## Introduction

Vancomycin is an old glycopeptide antimicrobial drug developed in the 1950s, which renders its pharmacological effects by interfering with the cell wall synthesis of Gram-positive bacteria (1). For decades, it remained in the first-line choice for the treatment of methicillin-resistant *Staphylococcus aureus* (MRSA), which was recommended by the guidelines issued by the Infectious Diseases Society of America and specific pediatric guidance (2). Vancomycin is a hydrophilic drug and mainly eliminated through the glomerular filtration. Approximately 90% of the drug remain unchanged during the excretion process (3). Thus, renal function plays a pivotal role in the pharmacokinetics of vancomycin.

Augmented renal function (ARC) is defined as enhanced renal elimination of solute as compared with an expected baseline. In the context of antibacterial therapy, ARC has the potential to result in subtherapeutic dosing, treatment failure, or selection of resistant microorganisms (4). One study suggested that a large proportion (67%) of critically ill children develop ARC during their stay at the intensive care unit (5). However, the mechanism of ARC has not reached a consensus, but several authors argued that systemic inflammatory response syndrome (SIRS) and renal function reserve (RFR) can be associated with ARC (6). We believe that the ARC infant population share similar physiological condition and possess their unique pharmacokinetics characteristics. Thus, it is important to establish population pharmacokinetics (PPK) models of vancomycin based on different renal function levels.

Several studies on the PPK model of vancomycin for neonates and infants have been published. However, findings from these reports varied a lot. The full characteristics of these previous results are listed in Table 1. We found that almost all of these models included weight (WT) as a covariate in the final model, but some renal function indicators, like serum creatinine (SCR) or creatinine clearance rate (CLCR), were only retained in part of these models. One of explanations is that all of these PPK models were commonly built on a mixture of patients with different renal function levels. Due to population selecting differences, some factors that should be included or excluded in certain group of people with similar physiological condition may or may not be retained in the models, which led to some missing parameters or redundancy of some parameters.

**Table 1:** A mini-review of previous vancomycin PPK models for pediatric patients.

**Modeling group**	**Study size**	**Age range**	**Final model**	**Authors**
**Models included renal function indicators (CLCR, SCR, and eGFR) as a covariate**				
Chinese children with malignant hematological disease	70	0.3–17.7 years	$\text{CL}\left(\text{L}/\text{h}\right)=4.37\times {\left[\frac{\text{WT}\left(\text{kg}\right)}{20.2}\right]}^{0.677}\times {\left[\frac{\text{CLCR}\left(\text{ml}/\text{min}/1.73\;{\text{m}}^{2}\right)}{191}\right]}^{1.03};\;$ $\text{V}\left(\text{L}\right)=119\times {\left[\frac{\text{WT}\left(\text{kg}\right)}{20.2}\right]}^{0.838}\;$	Zhao et al. (24)
Chinese neonatal intensive care unit patients	213	PMA: 28–47.9 weeks	$\text{CL}\left(\text{L}/\text{h}\right)=4.87\times {\left[\frac{\text{WT}\left(\text{kg}\right)}{70}\right]}^{0.75}\times \left[\frac{{\text{PMA}\left(\text{week}\right)}^{4.61}}{{\text{PMA}\left(\text{week}\right)}^{4.61}+34.5^{4.61}}\right]\times {\left[\frac{\text{SCR}\left(\text{mg}/\text{dL}\right)}{0.28}\right]}^{-0.221};\;$ $\text{V}\left(\text{L}\right)=40.7\times \frac{\text{WT}\left(\text{kg}\right)}{70}\;$	Chen et al. (1)
Chinese neonatal intensive care unit patients	80	PNA: 4–126 days	$\text{CL}\left(\text{L}/\text{h}\right)=0.309\times {\left[\frac{\text{WT}\left(\text{kg}\right)}{2.9}\right]}^{1.55}\times {\left[\frac{23.3}{\text{SCR}\left(\text{μmol}/\text{L}\right)}\right]}^{0.337};\;$ $\text{V}\left(\text{L}\right)=2.63\times {\left[\frac{\text{WT}\left(\text{kg}\right)}{2.9}\right]}^{1.05}\;$	Li et al. (21)
American pediatric cardiac surgical population	261	PMA: 42.6–76.9 weeks	$\text{CL}\left(\text{L}/\text{h}\right)=7.86\times {\left[\frac{\text{WT}\left(\text{kg}\right)}{70}\right]}^{0.75}\times {\left[\frac{\text{CLCR}\left(\text{mL}/\text{min}/1.73\;{\text{m}}^{2}\right)}{84}\right]}^{0.9}\times {\left[\frac{1}{1+{\left[\frac{\text{PMA}\left(\text{week}\right)}{50}\right]}^{-0.285}}\right]}^{0.795};\;$ $\text{V}\left(\text{L}\right)=63.6\times \frac{\text{WT}\left(\text{kg}\right)}{70}\;$	Moffett et al. (25)
American pediatric ventricular assist device population	69	2.4–11.9 years	$\text{CL}\left(\text{L}/\text{h}\right)=4.35\times {\left[\frac{\text{WT}\left(\text{kg}\right)}{70}\right]}^{0.75}\times 0.33^{LN\left[\frac{SCR\left(mg/dL\right)}{0.58}\right]};\;$ $\text{V}\left(\text{L}\right)=87.8\times \frac{\text{WT}\left(\text{kg}\right)}{70}\;$	Moffett et al. (26)
Korean neonatal intensive care unit neonates	207	PMA: 24–48.4 weeks	$\text{CL}\left(\text{L}/\text{h}\right)=2.09\times {\left[\frac{\text{WT}\left(\text{kg}\right)}{70}\right]}^{0.75}\times {\left[\frac{\text{PMA}\left(\text{week}\right)}{31.7}\right]}^{0.795}\times {\left[\frac{CLCR\left(mL/min/1.73\;m^{2}\right)}{50.3}\right]}^{0.741};\;$ $\text{V}\left(\text{L}\right)=45.6\times \frac{\text{WT}\left(\text{kg}\right)}{70}\;$	Lee et al. (27)
**Models not included renal function indicators as a covariate**				
Spanish neonates	70	PMA: 25.1–48.1 weeks	CL(L/h) = [0.00192 × PMA(week) × (1 + 0.65 × *AMX*)] × WT(kg); V(L) = [0.572 × (1 − 0.344 × *SPI*)] × *WT*(*kg*)	Marqués-Miñana et al. (28)
French pediatric patients with solid or hematological tumor disease	121	Hematological malignancies: mean 9.1 years; solid malignancies: mean 7.1 years	$\text{CL}\left(\text{L}/\text{h}\right)=\theta_{CL}\times {\left[\frac{\text{WT}\left(\text{kg}\right)}{70}\right]}^{0.75};\;$ V(L) = 34.7	Guilhaumou et al. (29)
American children with cystic fibrosis	67	Mean age: 12.1 ± 5.3 years	$\text{CL}\left(\text{L}/\text{h}\right)=5.57\times {\left[\frac{\text{WT}\left(\text{kg}\right)}{70}\right]}^{0.75};\;$ $\text{V}\left(\text{L}\right)=44.1\times \frac{\text{WT}\left(\text{kg}\right)}{70}\;$	Stockmann et al. (30)
Chinese neonates and young infants	316	PNA: 2–77 days	$\text{CL}\left(\text{L}/\text{h}\right)=0.42\times {\left[\frac{\text{WT}\left(\text{kg}\right)}{3.22}\right]}^{0.888}\times {\left[\frac{\text{PNA}\left(\text{days}\right)}{29}\right]}^{0.449};\;$ Q(L/h) = 1.161	Song et al. (31)
Chinese infants	61	PNA: 0.003–0.97 years	$\text{CL}\left(\text{L}/\text{h}\right)=0.449\times e^{0.0193}\times {\left[\frac{\text{WT}\left(\text{kg}\right)}{3.22}\right]}^{0.0643}\times {\left[\frac{\text{PNA}\left(\text{year}\right)}{0.1}\right]}^{0.289};\;$ V(L) = 4.45	Sheng et al. (32)
Chinese hematologic malignancy with augmented renal clearance children	53	2.24–17.87 years	$\text{CL}\left(\text{L}/\text{h}\right)=6.32\times {\left[\frac{\text{WT}\left(\text{kg}\right)}{70}\right]}^{0.75};\;$ $\text{V}\left(\text{L}\right)=39.6\times \frac{\text{WT}\left(\text{kg}\right)}{70}\;$	Lv et al. (7)

*θ_CL_ = 3.49, 4.66, 4.97 in patients managed for hematological malignancies with or without cyclosporine co-administration and for solid malignancies. CL, clearance; V, apparent volume of distribution; Q, fluid flow/intercompartmental clearance; WT, weight; PMA, postmenstrual age; SCR, serum creatinine; CLCR, creatinine clearance rate; PNA, postnatal age; AMX, amoxicillin–clavulanic acid; SPI, spironolactone*.

The aim of this study was to develop two PPK models of vancomycin for Chinese infants with normal and ARC levels and to analyze the parameter differences between the two models. We also wanted to determine whether the predictive performance of the two final models was better or not when compared with that of a third model built on the whole population data with all levels of renal function (a traditional approach used by previous studies).

## Materials and Methods

### Patients

Pediatric patients who received vancomycin treatment between January 2017 and July 2021 in Children's Hospital of Nanjing Medical University were enrolled in our study. The inclusion criteria were as follows: (1) post-natal age between 1 and 24 months, (2) received vancomycin through an intravenous infusion for at least 3 days, (3) at least one trough and one peak vancomycin blood concentrations were assayed, and (4) estimated glomerular filtration rate (eGFR) ≥ 30 ml/min/1.73 m^2^. Age, WT, height (HT), alanine transferase (ALT), aspartate transaminase (AST), blood urea nitrogen (BUN), SCR, Cystatin C (CYSC), albumin (ALB), total protein (TP), daily dosage (DD), and concomitant medication were retrospectively collected from the electronic medical records of the hospital information system. eGFR was calculated by the modified Schwartz formula (7):


$$eGFR=\frac{88.4\times k\times HT(cm)}{SCR(\mu mol/L)}$$

where ***k*** is 0.33 for preterm infants <1 year, 0.45 for full-term infants <1 year, and 0.55 for children between 2 and 12 years.

The external validation groups were randomly sampled from the whole population group. The rest of the patients were all recruited in the modeling group. According to Heilbron et al. normal GFR during infancy classification (8), we choose 30–86 ml/min/1.73 m^2^ as the threshold value for infant normal renal function level. Patients whose eGFR were between 30 and 86 ml/min/1.73 m^2^ were enrolled in the normal renal function group, while patients whose eGFR were ≥86 ml/min/1.73 m^2^ were enrolled in the ARC group. The study was conducted according to the guidelines of the Declaration of Helsinki and was approved by the Ethics Committee of the Children's Hospital of Nanjing Medical University (9). Patient consent was waived due to the nature of retrospective study.

Vancomycin (VIANEX S.A, Pallini, Greece) was administrated through intravenous infusion. For studied infants, the dosage of 40 mg/kg should be administrated two to four times per day, and the intravenous infusion time should be longer than 60 min. Dose adjustment was performed according to the results of therapeutic drug monitoring (TDM) of vancomycin, clinical efficacy, and adverse reactions.

### Bioassay

Whole blood samples were collected and assayed 30 min before the fifth administration of a series of same dosage of vancomycin for the trough concentration data and 30 min after the fifth administration of vancomycin for the peak concentration data. Enzyme multiplied immunoassay technique (Emit® 2000; SIEMENS, Munich, Germany) was employed for the quantitative analysis for vancomycin. The calibration range of the assay was 2.0–50 μg/ml (1.3–34 μmol/L). Quality control samples with a deviation of ±15% were applied to ensure the accuracy and precision of the EMIT method. The accuracy and precision of quality control samples based on three concentration levels were all within the acceptable criteria. At least one trough and one peak concentrations were collected for each enrolled child.

### Model Building

The NONMEN program (Version 7.4; Icon Inc., North Wales, PA, USA) compiled with gFortran (Version 4.9.2) was employed to establish the PPK models of vancomycin. R package version 3.6.1, and Xpose 4.5.3 were used to evaluate the models (10). The following model building procedure was employed to develop three models in parallel:

Model 1: Vancomycin PPK model for normal renal function (eGFR between 30 and 86 ml/min/1.73 m^2^) group
Model 2: Vancomycin PPK model for ARC (eGFR ≥ 86 ml/min/1.73 m^2^) group
Model 3: Vancomycin PPK model for the whole population group with all levels of renal function (the patient data model 3 built was generated by combining the patient data for models 1 and 2).

### Base Model

According to previous PPK studies of vancomycin in pediatric patients, we chose one-compartment model with first-order elimination, specified to NONMEM by ADVAN1-TRANS2 subroutine, as the foundation of base model. The one-compartment PK parameters include clearance (CL) and apparent volume of distribution (V). Between-subject variability (BSV) was described by exponential, proportional, and additive models separately; and exponential model was chosen at last for its better fitting results. The formula is described as follows:


$$\begin{array}{l} {\text{P}}_{\text{i}}=\text{T}\text{V}\left(\text{P}\right)\times {\text{e}}^{\eta_{\text{i}}} \end{array}$$

where ***P***_***i***_ represents the ith patient's individual PK parameter value; ***TV(P***) represents the typical individual parameter value; **η**_***i***_ is the random variable, which has zero mean and variance of ω^2^; and ***EXP***(**η**_***i***_) represents the deviation of ***TV***(***P***) from ***P***_***i***_.

Then, residual variance was evaluated by the additive model, proportional model, and mixed-error model, as follows:


$$\begin{array}{r} \text{Y}=\text{F}+\varepsilon \\ \text{Y}=\text{F}\times \left(1+\varepsilon \right)\\ \text{Y}=\text{F}\times \left(1+\varepsilon_{1}\right)+\varepsilon_{2} \end{array}$$

where ***Y*** represents the individual observed concentration and ***F*** represents the individual predictive concentration. **ε** is a symmetrical variable with a mean of zero and variability of σ^2^. Among all of three residual variance models, the one that can reach the smallest objective function values (OFVs) is retained in the base model. In addition, the final results of bootstrap analysis and the trend of goodness-of-fit (GOF) plots determined whether this expression of residual variance can be at last retained in the final model or require some modifications.

### Covariate Model

Previous studies have identified that WT and (or) age were the most important variables that must be taken into consideration when establishing PPK models for young infants (11). Thus, these two covariates were evaluated at first in a series of maturation models. Maturation refers to the process of becoming mature in individual traits, like personality and behavior, and is generally considered a continuous function that achieves an asymptote at the adult value at some growth point (12). Similarly, the maturation model that can reach the smallest OFV was recognized as the intermediate model and developed further. The general maturation model expresses as follows: where ***COV***_***median***_ means the median of the covariate, ***MF*** means the maturation factor:


$$\begin{array}{r} P_{i}=TV\left(P\right)\times {\left[\frac{COV}{{COV}_{median}}\right]}^{m}\times MF \end{array}$$

Maturation model I: ***MF*** was fixed to 1. Exponents ***m*** and ***n*** were unfixed.


$$\begin{array}{r} CL=TV\left(CL\right)\times {\left[\frac{WT}{{WT}_{median}}\right]}^{m}\;\left(I\right)\\ V=TV\left(V\right)\times {\left[\frac{WT}{{WT}_{median}}\right]}^{n}\;\left(I\right) \end{array}$$

Maturation model II: ***MF*** was fixed to 1. Exponent ***m*** was fixed to 0.75 empirically, and ***n*** was removed from the expression of ***V*** (12).


$$\begin{array}{r} CL=TV\left(CL\right)\times {\left[\frac{WT}{{WT}_{median}}\right]}^{0.75}\;\left(II\right)\\ V=TV\left(V\right)\times \frac{WT}{{WT}_{median}}\;\left(II\right) \end{array}$$

Maturation model III: Exponent ***m*** was fixed to 0.75 empirically. ***MF*** was calculated as the following equation, where ***TM***_**50**_ is the age at which ***CL*** maturation reaches 50% of that of adults and ***Hill*** is a slope parameter (13).


$$\begin{array}{l} CL=TV\left(CL\right)\times {\left[\frac{WT}{{WT}_{median}}\right]}^{0.75}\times MF,\;MF=\frac{1}{1+{\left[\frac{Age}{{TM}_{50}}\right]}^{Hill}}\;\left(III\right) \end{array}$$

Maturation model IV and V: **θ**_**0**_ was defined as an exponent at an ideal ***WT*** of 0 in maturation model IV or at an ***Age*** of 0 years in maturation model V. ***k***_***max***_ is the maximum decrease of the exponent; ***k***_**50**_ is the ***WT*** (maturation model IV) or ***Age*** (maturation model V) at which a 50% decrease in the maximum decrease is attained; and the ***Hill*** coefficient is used to determine the steepness of the sigmoid decline (13, 14).


$$\begin{array}{r} CL=TV\left(CL\right)\times {\left[\frac{WT}{{WT}_{median}}\right]}^{m},\;m=\theta_{0}-\frac{k_{\max}\times {WT}^{Hill}}{{k_{50}}^{Hill}+{WT}^{Hill}}\;\left(IV\right)\\ CL=TV\left(CL\right)\times {\left[\frac{WT}{{WT}_{median}}\right]}^{m},\;m=\theta_{0}-\frac{k_{\max}\times {Age}^{Hill}}{{k_{50}}^{Hill}+{Age}^{Hill}}\;\left(V\right) \end{array}$$

Once a proper maturation model was selected, the intermediate PPK model was further developed by stepwise forward addition and backward exclusion method. In these two consecutive procedures, sex, age, HT, ALT, AST, BUN, SCR, CYSC, ALB, TP, eGFR, and concomitant medications were screened. The covariate screening criteria were as follows: (1) in the stepwise forward addition process, if OFV decreased >3.84 (χ^2^, *df* = 1, *p* < 0.05) after the inclusion of a candidate covariate, then this covariate can be retained in the model. (2) In the backward exclusion process, if the increase in OFV was <10.83 (χ^2^, *df* = 1, *p* < 0.001) after the exclusion of an in-model covariate, then the covariate was excluded from the final model.

### Model Evaluation

GOF plots, which consist of observation (DV) vs. individual prediction (IPRED), DV vs. population prediction (PRED), conditional weighted residual errors (CWRES) vs. time, and CWRES vs. PRED, were utilized to visually check the performance of the final models (15). Visual predictive check (VPC) was also employed to check the predictive ability of each model. Then, bootstrap analysis was employed to check the stability of the final parameter estimates with the repetition of 1,000 NONMEM runs of the final models (16). Success rate of 1,000 NONMEN runs, 2.5–97.5% range of the bootstrap results, and bias between bootstrap results and NONMEN estimates was calculated to quantitatively evaluate the accuracy of parameter estimates. Finally, an external validation was carried out for all the final models to test and compare the predictive ability of each model. Model 1 and Model 3 were compared among a group of external infants with normal renal functions (eGFR between 30 and 86 ml/min/1.73 m^2^), while Model 2 and Model 3 were compared among a group of external infants with ARCs (eGFR ≥ 86 ml/min/1.73 m^2^). The mean prediction error (MPE), mean relative prediction error (MPE%), mean absolute prediction error (MAE), mean relative absolute prediction error (MAE%), and root mean squared prediction error (RMSE) suggested the predictive precision of the final models (17). These indicators were calculated by the following equations (18):


$$\begin{array}{l} MPE=\frac{1}{n}\overset{n}{\underset{j=1}{\sum }}\left(C_{{IPRED}_{j}}-C_{{OBS}_{j}}\right);MAE=\frac{1}{n}\overset{n}{\underset{j=1}{\sum }}|C_{{IPRED}_{j}}-C_{{OBS}_{j}}|;\; \end{array}$$

$$\begin{array}{l} MPE\%=\frac{1}{n}\overset{n}{\underset{j=1}{\sum }}\left(\frac{C_{{IPRED}_{j}}-C_{{OBS}_{j}}}{C_{{OBS}_{j}}}\right);\;MAE\%=\frac{1}{n}\overset{n}{\underset{j=1}{\sum }}|\frac{C_{{IPRED}_{j}}-C_{{OBS}_{j}}}{C_{{OBS}_{j}}}|; \end{array}$$

$$\begin{array}{l} RMSE=\sqrt{\frac{1}{n}\overset{n}{\underset{j=1}{\sum }}{\left(C_{{IPRED}_{j}}-C_{{OBS}_{j}}\right)}^{2}} \end{array}$$

where ***n*** represents the number of observations, ***C***_***IPRED***_ represents individual predictive concentration, and ***C***_***OBS***_ represents observed concentration. Statistical analysis was performed by SPSS 22.0 statistical software (IBM, Armonk, NY, USA). Wilcoxon signed-rank test were used to compare the external validation results.

## Results

### Patients

A total of 115 patients in total were enrolled in the modeling group, among whom 61 patients were enrolled in the normal renal function group and 64 patients were enrolled in the ARC group. The numbers of the two groups add up over 115 because some patients experienced renal function fluctuation during long treatment period and were counted twice in both groups. In total, 276 vancomycin concentrations were assayed, including 158 trough concentrations and 118 peak concentrations. More detailed demographic, laboratory, and clinical data for each group are summarized in Table 2.

**Table 2:** Demographic, laboratory and clinical data of the modeling groups.

**Variable**	**Reduced renal function group (Model 1)**		**Normal renal function group (Model 2)**		**Whole population group (Model 3)**	
	**Mean values ± SD**	**Median (range)**	**Mean values ± SD**	**Median (range)**	**Mean values ± SD**	**Median (range)**
Number of patients (male/female)	61 (37/24)	/	64 (41/23)	/	115 (73/42)	/
Number of observations (trough/peak)	135 (69/66)	/	139 (88/51)	/	276 (158/118)	/
Age, months	2.31 ± 3.96	1 (1–24)	3.36 ± 4.75	1 (1–24)	2.87 ± 4.45	1 (1~24)
Number of preterm infants	29	/	17	/	46	/
WT, kg	2.86 ± 2.16	2.25 (1.15–13)	5.34 ± 2.71	4.60 (2.2–14)	4.11 ± 2.79	3.3 (1.15~14)
HT, cm	45.84 ± 10.81	44 (32–90)	56.57 ± 11.80	54 (42.9–110)	51.73 ± 12.86	50 (32~110)
ALT, U/L	57.14 ± 134.67	19.50 (2–858)	61.55 ± 97.37	30 (6–722)	62.97 ± 126.24	24 (2~722)
AST, U/L	105.62 ± 314.04	36 (7–2949)	67.78 ± 95.74	40 (11.1–751)	84.26 ± 228.54	38 (7~2949)
BUN, mmol/L	4.73 ± 5.75	3.44 (0.7–52)	3.03 ± 1.53	2.8 (0.4–8.3)	3.84 ± 4.22	2.96 (0.4~25.3)
SCR, μmol/L	30.80 ± 32.27	27.1 (14–315)	16.84 ± 4.17	16.8 (8–29)	23.54 ± 23.63	18.95 (8~147)
CYSC, mg/L	1.56 ± 0.48	1.51 (0.03–3.18)	1.35 ± 0.45	1.37 (0.02–2.63)	1.45 ± 0.48	1.43 (0.02~2.98)
ALB, g/L	31.68 ± 6.13	31.35 (7.1–58.1)	35.24 ± 3.35	35.1 (27.7–44.1)	33.43 ± 5.16	33.9 (7.1~45.5)
TP, g/L	48.02 ± 10.05	46.90 (10–83.1)	55.89 ± 6.55	55.1 (40.2–72.1)	51.87 ± 9.25	51.1 (10~81.2)
eGFR, ml/min/1.73 m^2^	58.78 ± 21.66	57.56 (30–85.56)	140.76 ± 35.70	128 (90.8–280)	99.44 ± 49.59	98.7 (30~280)
DD, mg/d	92.90 ± 61.85	75 (24–320)	224.30 ± 125.63	216 (50–640)	172.57 ± 126.43	145 (24–640)
Co-administration of meropenem or imipenem	47	/	54	/	91	/
Trough concentration, mg/L	8.29 ± 4.67	7.05 (2.1–22.2)	7.93 ± 4.66	7 (2.1–23)	8.31 ± 4.75	7.1 (2.1–23)
Peak concentration, mg/L	22.28 ± 8.75	19.9 (6.8–46.9)	18.82 ± 6.76	18.7 (6.1–36.6)	20.61 ± 7.67	19.1 (6.1–46.9)

*WT, weight; HT, height; ALT, alanine transferase; AST, aspartate transaminase; BUN, blood urea nitrogen; SCR, serum creatinine; CYSC, Cystatin C; ALB, albumin; TP, total protein; eGFR, estimated glomerular filtration rate; DD, daily dosage*.

There were 55 patients in total enrolled in the external validation group, and among them, 21 belonged to the reduced renal function group, and the rest 34 belonged to the normal renal function group. More detailed demographic, laboratory, and clinical data for each group are summarized in Table 3.

**Table 3:** Demographic, laboratory, and clinical data of the external validation groups.

**Variable**	**Normal renal function group**		**Augmented renal function group**	
	**Mean values ± SD**	**Median (range)**	**Mean values ± SD**	**Median (range)**
Number of patients (male/female)	21 (14/7)	/	34 (20/14)	/
Number of observations (trough/peak)	46 (23/23)	/	68 (34/34)	/
Age, months	1 ± 0	1 (1–1)	2.76 ± 3.42	1 (1–12)
Number of preterm infants	14	/	5	/
Weight, kg	2.48 ± 1.19	1.89 (1.37–5)	4.91 ± 2.25	4.20 (1.85–12)
Height, cm	44.50 ± 6.81	43 (32–57)	56.25 ± 9.48	52.50 (45–80)
ALT, U/L	35.52 ± 42.44	18 (2–189)	63.44 ± 124.81	25 (3–722)
AST, U/L	82.39 ± 126.68	44 (7–474)	65.87 ± 124.42	39.50 (10–751)
BUN, mmol/L	4.05 ± 1.69	4.18 (1.93–7.3)	2.42 ± 1.26	2.15 (0.92–6.66)
SCR, μmol/L	29.37 ± 9.58	26.50 (20.6–101)	17.69 ± 4.92	17.00 (11–33)
CYSC, mg/L	1.63 ± 0.42	1.57 (0.56–2.65)	1.39 ± 0.49	1.35 (0.04–2.63)
ALB, g/L	32.82 ± 5.23	33.7 (24.5–44.8)	35.96 ± 3.75	36.7 (22.9–44.4)
TP, g/L	47.90 ± 7.26	46.8 (38.3–64.5)	56.67 ± 7.52	56.85 (39.8–68.8)
eGFR, ml/min/1.73 m^2^	63.45 ± 11.28	63.33 (34.49–84.65)	137.34 ± 42.62	131.10 (88.89–311.82)
DD, mg/d	81.02 ± 48.55	60 (15–192)	202.25 ± 94.17	197.50 (50–440)
Co-administration of meropenem or imipenem	20	/	26	/
Trough concentration, mg/L	8.27 ± 4.17	6.80 (2.9–18.2)	6.01 ± 2.65	5.35 (2.7–14.4)
Peak concentration, mg/L	21.82 ± 7.26	19.94 (13.3–48.1)	22.33 ± 7.12	21.65 (9.1–46.8)

*WT, weight; HT, height; ALT, alanine transferase; AST, aspartate transaminase; BUN, blood urea nitrogen; SCR, serum creatinine; CYSC, Cystatin C; ALB, albumin; TP, total protein; eGFR, estimated glomerular filtration rate; DD, daily dosage*.

### Model Building

According to covariate screening criteria, for Model 1 and Model 3, SCR and WT were identified as significant covariates. However, for Model 2, only WT was kept in the final model. The establishment of intermediate maturation model and the covariate screening process of Model 1 were exhibited in Table 4 as an example. The parameter estimates are listed in Tables 5–7. The three final models are as follows:

**Table 4:** The covariate model building process of Model 1.

**Model**	**Description**	**OFV**	**ΔOFV**	***p*-Value**	**Reserve**
Base model	One-compartment model with first-order elimination	715.776	0	/	/
**Maturation model selecting process**					
Maturation model I	See *Covariate Model* section: equation I	632.709	−83.067	<0.05	Yes
Maturation model II	See *Covariate Model* section: equation II	637.799	−77.977	<0.05	No
Maturation model III	See *Covariate Model* section: equation III	663.949	−51.827	<0.05	No
Maturation model IV	See *Covariate Model* section: equation IV	646.078	−69.698	<0.05	No
Maturation model V	See *Covariate Model* section: equation V	671.471	−44.305	<0.05	No
**Stepwise forward addition Round 1**					
Model A	Add co-administration of meropenem or imipenem on CL in maturation model I	631.345	−1.364	>0.05	No
Model B	Add SEX on CL in maturation model I	631.858	−0.851	>0.05	No
Model C	Add AGE on CL in maturation model I	632.462	−0.247	>0.05	No
Model D	Add HT on CL in maturation model I	628.091	−4.618	<0.05	Yes
Model E	Add ALT on CL in maturation model I	632.706	−0.003	>0.05	No
Model F	Add AST on CL in maturation model I	632.708	−0.001	>0.05	No
Model G	Add BUN on CL in maturation model I	626.547	−6.162	<0.05	Yes
Model H	Add SCR on CL in maturation model I	611.910	−20.799	<0.05	Yes
Model I	Add CYSC on CL in maturation model I	627.555	−5.154	<0.05	Yes
Model J	Add ALB on CL in maturation model I	632.315	−0.394	>0.05	No
Model K	Add TP on CL in maturation model I	632.552	−0.157	>0.05	No
Model L	Add eGFR on CL in maturation model I	620.829	−11.88	<0.05	Yes
**Stepwise forward addition Round 2**					
Model M	Add BUN on CL in model H	610.667	−1.243	>0.05	No
Model N	Add eGFR on CL in model H	606.684	−5.226	<0.05	Yes
Model O	Add HT on CL in model H	606.057	−5.853	<0.05	Yes
Model P	Add CYSC on CL in model H	609.149	−2.761	>0.05	No
**Stepwise forward addition Round 3**					
Model Q	Add eGFR on CL in model O	603.087	−2.97	>0.05	No
**Backward exclusion**					
Model R	Remove WT on CL and V from model O	688.498	82.441	<0.001	Yes
Model S	Remove SCR on CL from model O	628.091	22.034	<0.001	Yes
Model T	Remove HT on CL from model O	611.91	5.853	>0.001	No

*OFV, objective function value*.

**Table 5:** NONMEN estimates and bootstrap analysis of Model 1.

**Parameter**	**NONMEM estimate**	**RSE (%)**	**Bootstrap median**	**2.5%**~**97.5%**	**Bias (%)**
CL (L/h) = θ_1_*[WT (kg)/2.25]**θ_3_*e**[θ_5_*SCR (μmol/L)/27.1]; V(L) = θ_2_*[WT (kg)/2.25]**θ_4_					
θ_1_	0.407	9.3	0.3985	0.287~0.483	−2.13
θ_2_	1.86	8.4	1.85	1.58~2.17	−0.54
θ_3_	1.24	7.6	1.24	1.03~1.43	0
θ_4_	1.28	13.3	1.25	0.818~1.45	−2.4
θ_5_	−0.533	15.0	−0.5265	−0.671~-0.199	−1.23
BSV_CL	0.315	36.5	0.304	0.159~0.392	−3.62
PROP_RV	0.319	17.9	0.312	0.261~0.382	−2.24

*Success rate of 1,000 times bootstrap analysis: 100%. Bias (%) = (Bootstrap Median – NONMEM Estimate)/NONMEM estimate × 100%. RSE (%), relative standard error; BSV_CL, between-subject variability of clearance; PROP_RV, proportional residual variance*.

**Table 6:** NONMEN estimates and bootstrap analysis of Model 2.

**Parameter**	**NONMEM estimate**	**RSE (%)**	**Bootstrap median**	**2.5%**~**97.5%**	**Bias (%)**
CL (L/h) = θ_1_*[WT (kg)/4.6]**θ_3_; V (L) = θ_2_*[WT (kg)/4.6]**θ_4_					
θ_1_	0.756	5.2	0.755	0.684~0.834	−0.13
θ_2_	4.89	8.8	4.81	4.12~5.89	−1.64
θ_3_	1.03	13.1	1.04	0.7549~1.32	0.97
θ_4_	0.918	17.8	0.888	0.585~1.29	−3.27
BSV_CL	0.312	27.5	0.3015	0.202~0.378	−3.37
PROP_RV	0.319	16.5	0.326	0.272~0.381	−2.19

*Success rate of 1,000 times bootstrap analysis: 100%. Bias (%) = (Bootstrap Median – NONMEM Estimate)/NONMEM estimate × 100%. RSE (%), relative standard error; BSV_CL, between-subject variability of clearance; PROP_RV, proportional residual error variability*.

**Table 7:** NONMEN estimates and bootstrap analysis of Model 3.

**Parameter**	**NONMEM estimate**	**RSE (%)**	**Bootstrap median**	**2.5%**~**97.5%**	**Bias (%)**
CL (L/h) = θ_1_*[WT (kg)/3.45]**θ_3_*e**[θ_5_*SCR (μmol/L)/19]; V (L) = θ_2_*[WT (kg)/3.45]**θ_4_					
θ_1_	0.707	7.1	0.702	0.585~0.809	−0.71
θ_2_	3.39	7	3.39	2.96~3.85	0.00
θ_3_	1.23	5.3	1.23	1.09~1.36	0.00
θ_4_	1.29	8.1	1.28	1.06~1.45	−0.78
θ_5_	−0.377	13	−0.375	−0.469~-0.216	−0.53
BSV_CL	0.311	22.9	0.305	0.225~0.37	−1.93
PROP_RV	0.335	11.1	0.332	0.295~0.37	−0.90

*Success rate of 1,000 times bootstrap analysis: 100%. Bias (%) = (Bootstrap Median – NONMEM Estimate)/NONMEM estimate × 100%. RSE (%), relative standard error; BSV_CL, between-subject variability of clearance; PROP_RV, proportional residual variance*.

Model 1: The PPK model of vancomycin for Chinese infants with normal renal function.


$$\begin{array}{r} CL\left(L/h\right)=0.407\times {\left[\frac{WT\left(kg\right)}{2.25}\right]}^{1.24}\times e^{\frac{-0.533\times SCR\left(\mu mol/L\right)}{27.1}}\\ V\left(L\right)=1.86\times {\left[\frac{WT\left(kg\right)}{2.25}\right]}^{1.28} \end{array}$$

Model 2: The PPK model of vancomycin for Chinese infants with ARC.


$$\begin{array}{r} CL\left(L/h\right)=\;0.756\times {\left[\frac{WT\left(kg\right)}{4.6}\right]}^{1.03}\\ V\left(L\right)=\;4.89\times {\left[\frac{WT\left(kg\right)}{4.6}\right]}^{0.918} \end{array}$$

Model 3: The PPK model of vancomycin for the whole population with all levels of renal function.


$$\begin{array}{r} CL\left(L/h\right)=0.707\times {\left[\frac{WT\left(kg\right)}{3.45}\right]}^{1.23}\times e^{\frac{-0.377\times SCR\left(\mu mol/L\right)}{19}}\\ V\left(L\right)=\;3.39\times {\left[\frac{WT\left(kg\right)}{3.45}\right]}^{1.29} \end{array}$$

### Model Evaluation and Comparison

**Figure 1:**
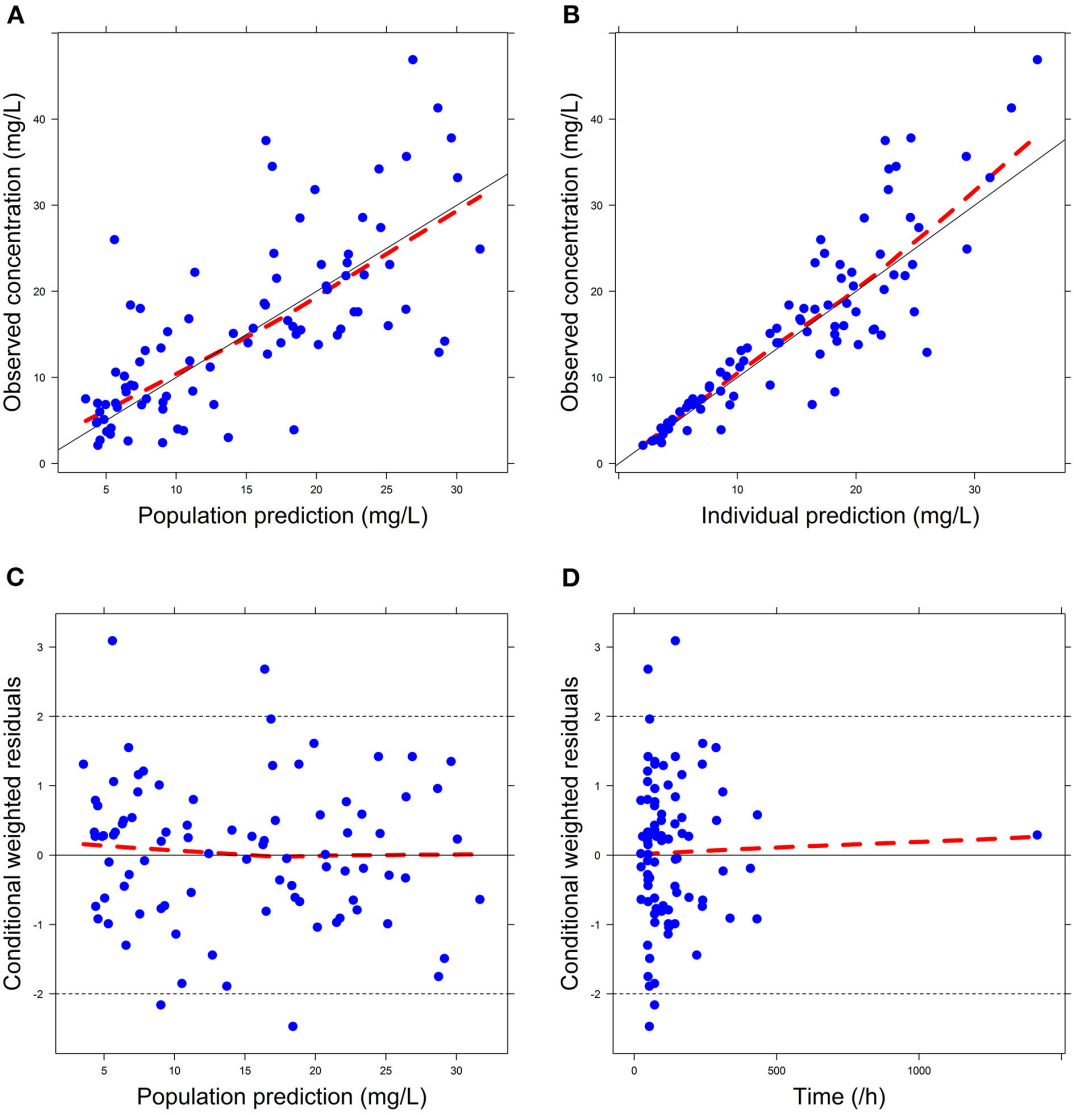
Goodness-of-fit (GOF) plots of Model 1: **(A)** observed concentration (DV) vs. population prediction (PRED); **(B)** DV vs. individual prediction (IPRED); **(C)** conditional weighted residual errors (CWRES) vs. PRED; **(D)** CWRES vs. Time.

**Figure 2:**
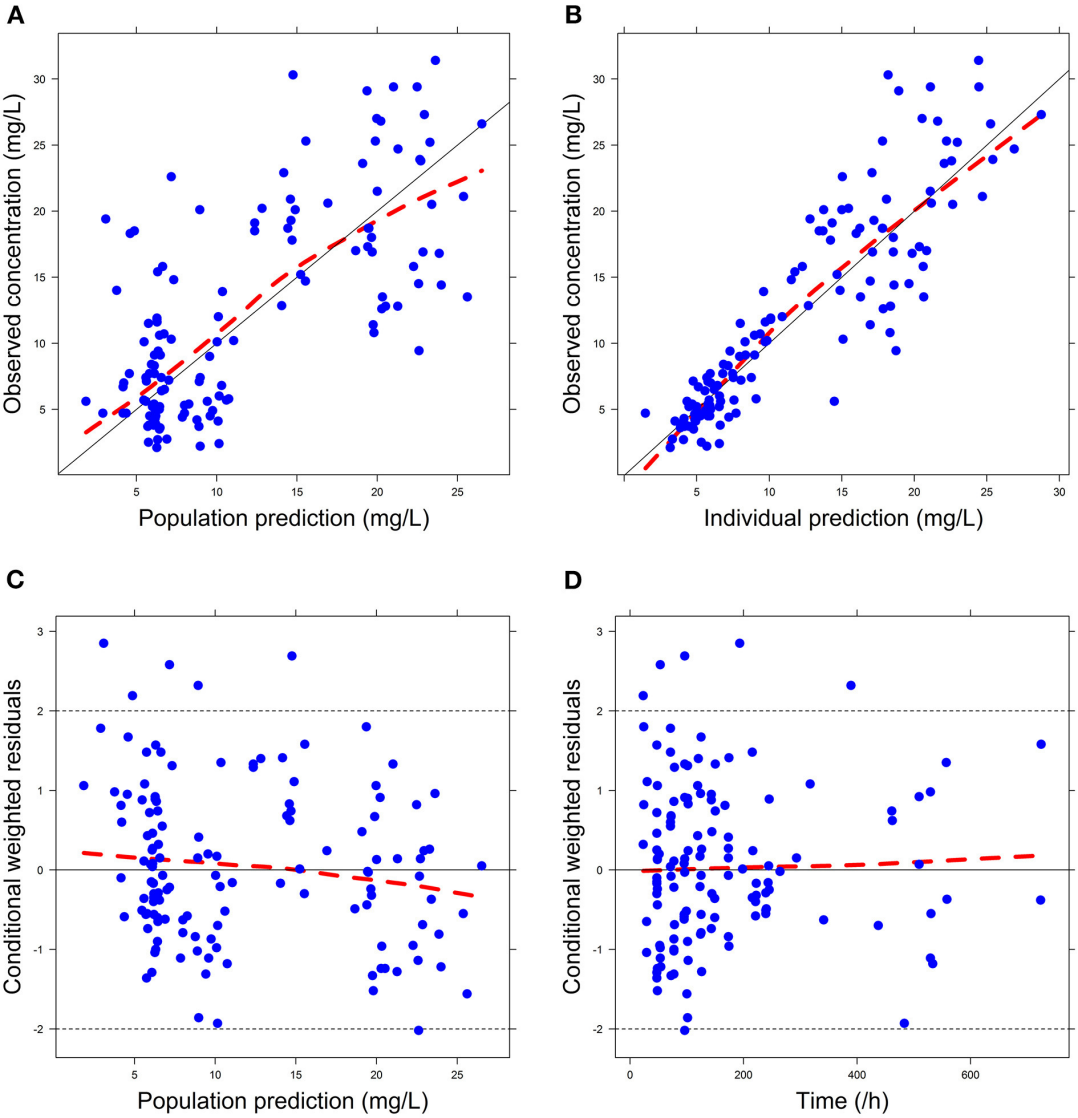
Goodness-of-fit (GOF) plots of Model 2: **(A)** observed concentration (DV) vs. population prediction (PRED); **(B)** DV vs. individual prediction (IPRED); **(C)** conditional weighted residual errors (CWRES) vs. PRED; **(D)** CWRES vs. Time.

**Figure 3:**
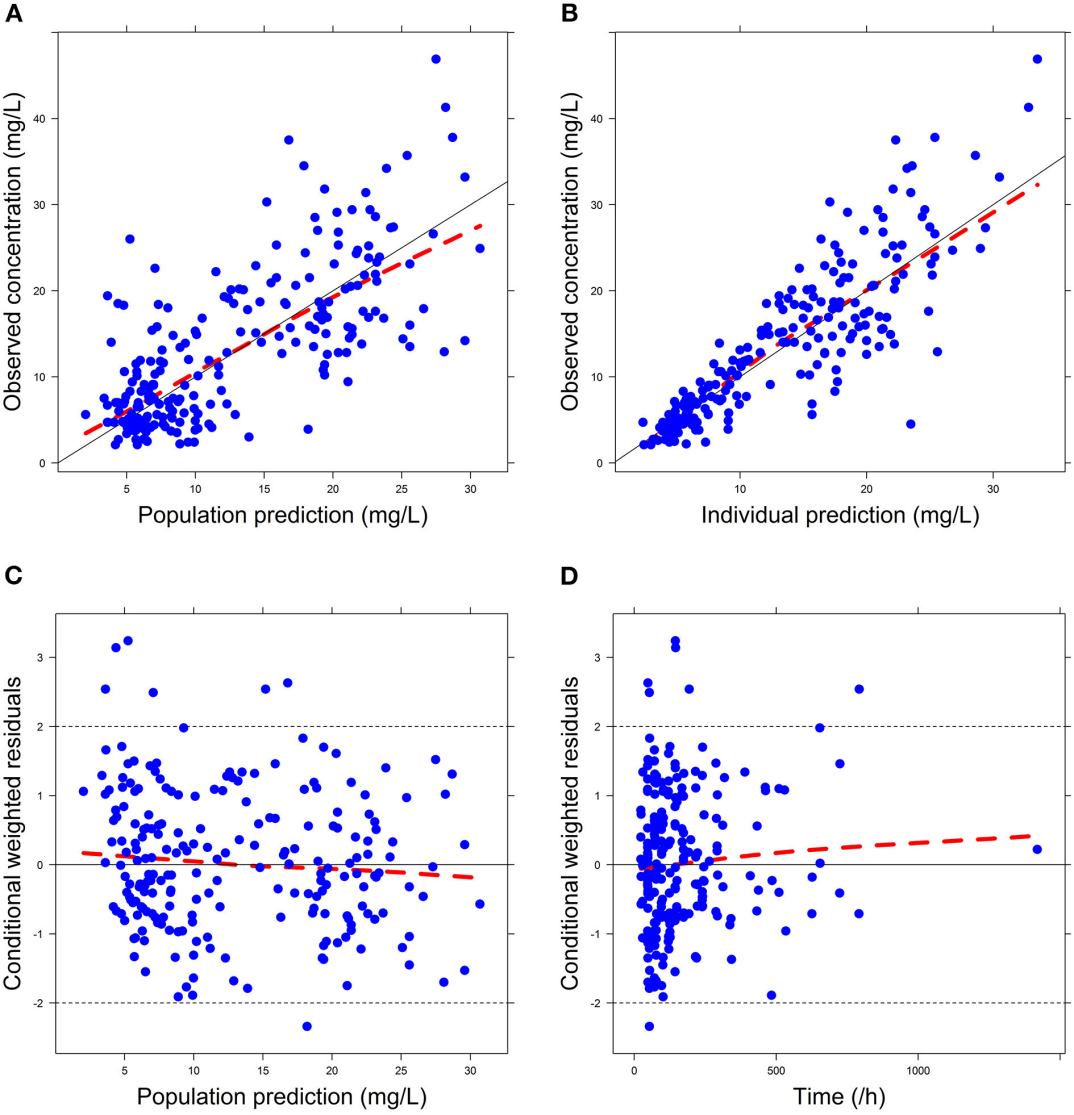
Goodness-of-fit (GOF) plots of Model 3: **(A)** observed concentration (DV) vs. population prediction (PRED); **(B)** DV vs. individual prediction (IPRED); **(C)** conditional weighted residual errors (CWRES) vs. PRED; **(D)** CWRES vs. Time.

**Figure 4:**
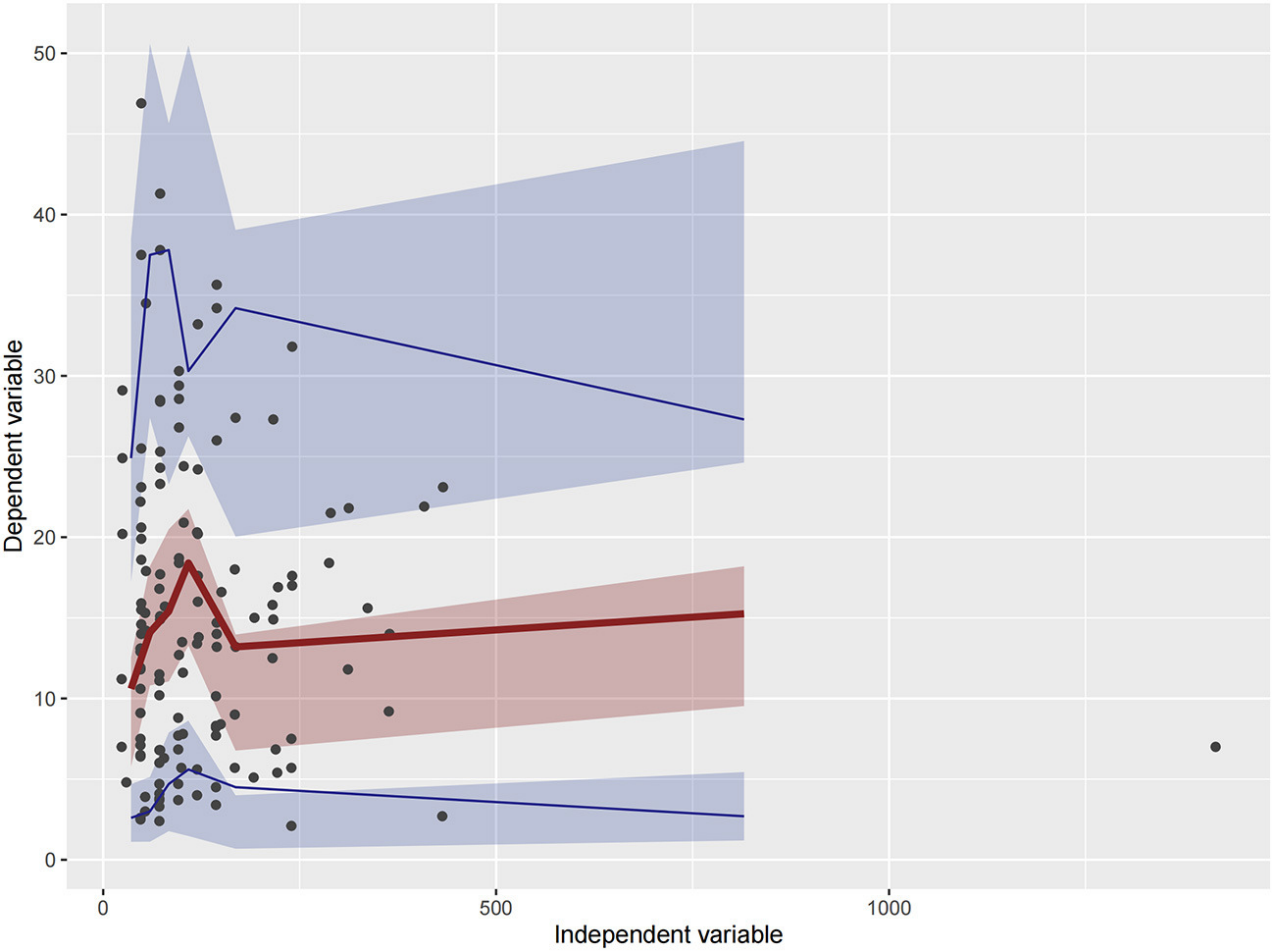
Visual predictive check (VPC) of Model 1. Circles represent observations. The red solid lines represent the median of simulated concentrations, and the blue solid lines represent the 90% PI (5 and 95%) of the predictive vancomycin concentrations. The shaded areas represent the 95% CI for each line.

**Figure 5:**
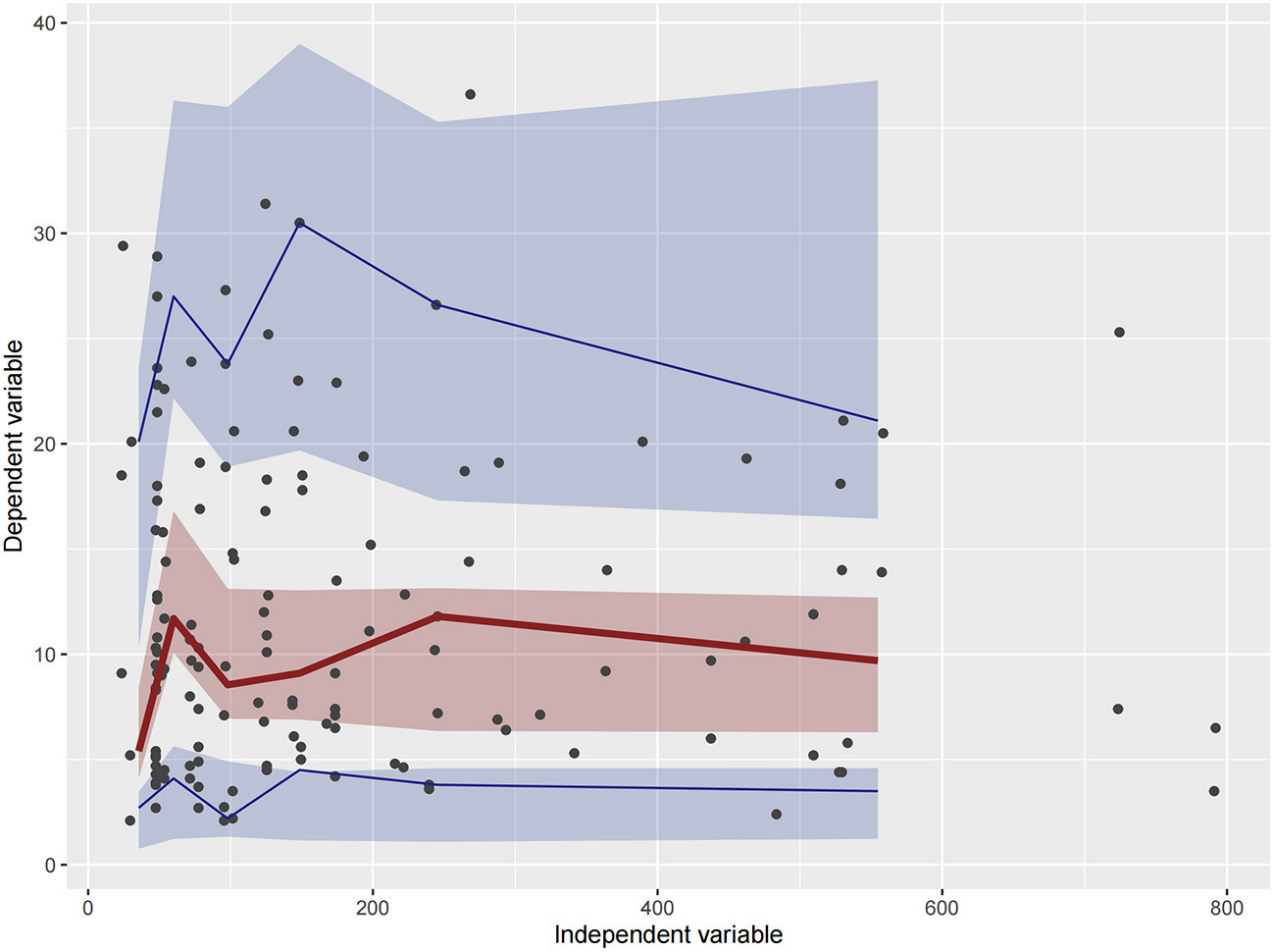
Visual predictive check (VPC) of Model 2. Circles represent observations. The red solid lines represent the median of simulated concentrations, and the blue solid lines represent the 90% PI (5 and 95%) of the predictive vancomycin concentrations. The shaded areas represent the 95% CI for each line.

**Figure 6:**
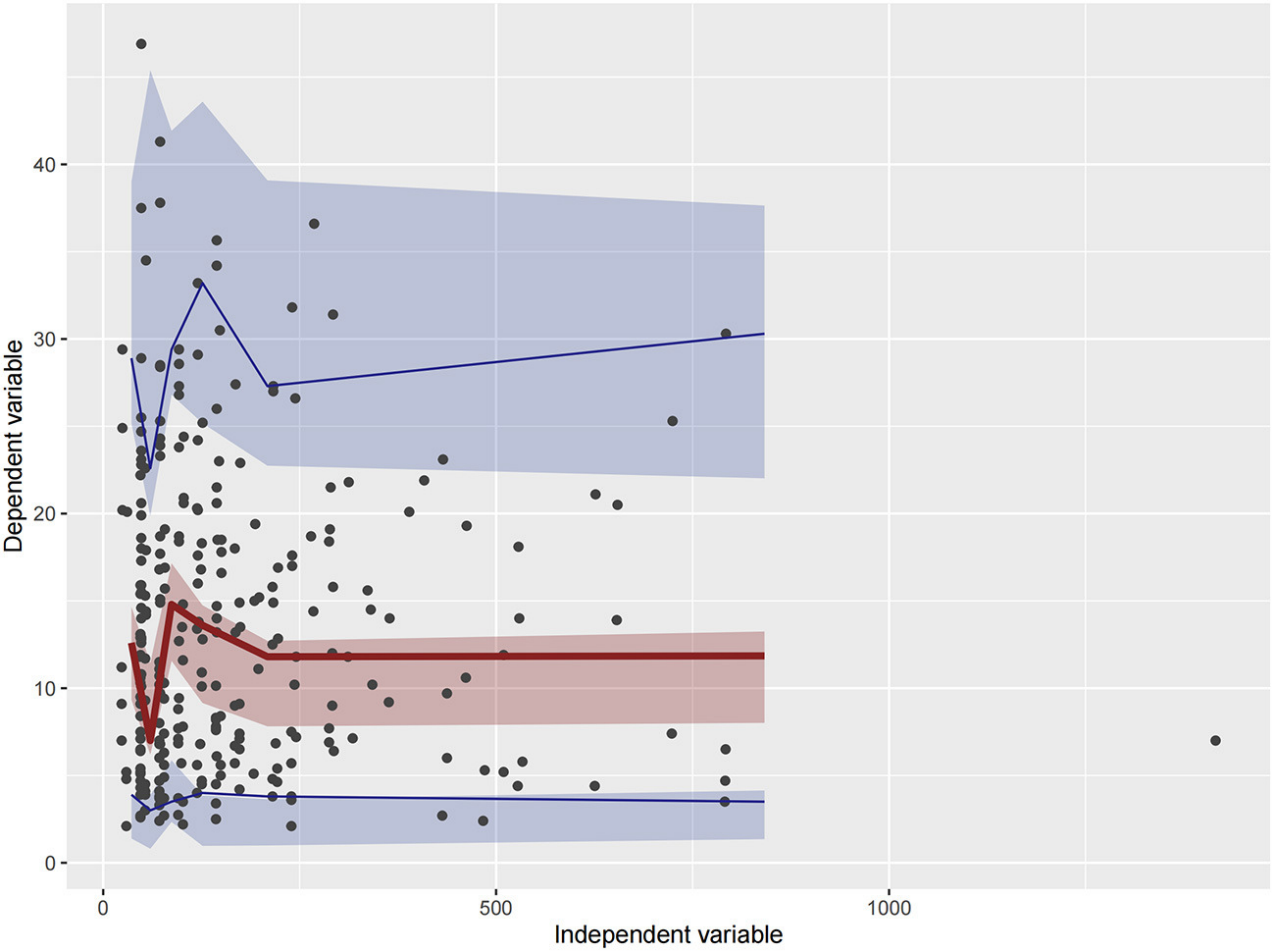
Visual predictive check (VPC) of Model 3. Circles represent observations. The red solid lines represent the median of simulated concentrations, and the blue solid lines represent the 90% PI (5 and 95%) of the predictive vancomycin concentrations. The shaded areas represent the 95% CI for each line.

Firstly, all three final models were inspected using GOF plots. Figures 1–3 suggest that the three final models showed no obvious bias or significant trends that are deviated from y = x or y = 0. The CWRES were randomly distributed around zero line, and most of the residuals range from −2 to 2. All these plots indicated the robustness of the final models. Figures 4–6, VPC results of the three models, suggest that the fifth and 95th (90%) percentile PIs covered most of the observations, indicating a good predictive performance of the final models. Then, bootstrap analysis was conducted for each model, and the results (Tables 5–7) suggest that the parameter estimates were close to the bootstrap results with all biases <5%. In addition, the success rate of 1,000 times bootstrap analysis was 100%, suggesting that all three models were stable. The results of shrinkage of empirical Bayes estimates (EBEs) of Model 1, Model 2, and Model 3 are listed in Table 8, which are all below 20%. Finally, the results of external validation are listed in Table 9. The external validation results indicated that the predictive performance of Model 1 and Model 2 are comparable with Model 3 with no statistical differences (*p* > 0.05), but not improved.

**Table 8:** Shrinkage of empirical Bayes estimates (EBEs) of Model 1, Model 2, and Model 3.

**Parameter**	**Model 1**	**Model 2**	**Model 3**
BSV_CL	10%	11%	11%
PROP_RV	16%	15%	14%

*BSV_CL, between-subject variability of clearance; PROP_RV, proportional residual variance*.

**Table 9:** The MPE, MPE%, MAE%, and RMSE values of the external validation.

**Validation group**	**Compared models**	**MPE**	**MPE%**	**MAE**	**MAE%**	**RMSE**	**Statistical difference**
Reduced renal function	Model 3	0.7334	18.49%	3.834	34.46%	6.101	No
	Model 1	0.9682	18.80%	3.913	33.87%	6.218	
Normal renal function	Model 3	−2.044	6.330%	3.817	31.75%	5.647	No
	Model 2	−2.415	2.058%	3.807	29.40%	5.706	

*MPE, mean prediction error; MPE%, mean relative prediction error; MAE, mean absolute error; MAE%, mean relative absolute error; RMSE, root mean square error*.

## Discussion

A good number of PPK models of vancomycin for Chinese pediatric patients have been established. However, to the best of our knowledge, the PPK model of vancomycin for Chinese infants based on different levels of renal function has not been proposed before. Zaric et al. established a PPK model of vancomycin for adult patients with different renal function levels in 2018 (19), but this method has not been investigated among Chinese infants yet. So this study was the first one to conduct such an attempt. Furthermore, after literature research, we found out that almost all PPK models of vancomycin for pediatric patients included body weight as an important covariate, but some typical physiological indicators that reflect renal function levels, such as SCR, CLCR, and eGFR, were not included in the final models. We speculated that this was because in certain group of people, out of some physiological reasons, these renal function indicators cannot synchronously reflect the change of blood vancomycin concentration, which eventually leads to the exclusion of these renal function indicators from the final models. Plus, we believe renal function level was the key factor that can define population group, whose data will later shape the PK characteristics of each final model. All of these findings and speculations led us to carry out such a study.

This study separately established the PPK models of vancomycin for Chinese infants with normal renal function and with ARC for the first time and compared the predictive ability of these two models with the whole population group model. In the model covariate optimization procedure, sex, age, WT, HT, ALT, AST, BUN, SCR, CYSC, ALB, TP, and eGFR were investigated. Among the pediatric population with normal renal function, WT and SCR are the main determinants for the vancomycin CL, which was in accordance with the model based on the whole population data. However, among the ARC group, only WT was included as a covariate, which agreed with the findings of Lv et al. in 2020, who demonstrated that body weight with allometric scaling was the only significant determinant on CL and V in the group of Chinese hematologic malignancy children with eGFR ≥130 ml/min/1.73 m^2^ (7), and Yamamoto et al. in 2009 observed that the clearance of vancomycin was linearly correlated to CLCR in patients with renal insufficiency (CLCR < 85 ml/min/1.73 m^2^), but there was no linear relationship when CLCR was ≥85 ml/min/1.73 m^2^ (20).

The diagnostic GOF plots, VPC, and bootstrap analysis indicated that all three models were stable. The external validation indicated not only the good predictive performance of the three models but also that the predictive performance of Model 1 and Model 2 is comparable with Model 3 with no statistical differences (*p* > 0.05). Additionally, Li et al. proposed a PPK model of vancomycin for Chinese ICU neonates, whose postnatal age ranged from 4 to 126 days, in 2018 (21). This was the latest model for this population prior to our study, and our model expanded the postnatal age range to 2 years.

The model for the normal renal function group included SCR as a covariate, but the model for the ARC group did not. Since vancomycin is hydrophilic with a large molecular volume and many non-ionic groups (22), the tissue distribution speed of vancomycin is expected to be limited by the lipid membrane. Moreover, Du et al., by using an established physiologically based pharmacokinetics (PBPK) model, found out that the concentration of vancomycin in renal tubules was about 40–50 times higher than that in plasma. Also, the PK characteristics of vancomycin in kidney were quite different from that in plasma and renal tubules, which showed a delay in time to peak and a much slower drug elimination speed. In the PBPK model of kidney-injury patients, they found out that concentrations of vancomycin in both the kidney tubule and the kidney decreased slightly. When the renal function of the patients changed from moderate to severe injury, both the amount of vancomycin and its concentration in kidney tubules and kidney decreased further (23).

Thus, one of the possible explanations for the difference between Models 1 and 2 is that among the pediatric population with normal renal function, due to less permeability of lipid membrane compared with the ARC population, it is a longer process for both SCR and vancomycin to be transported from plasma to kidney. So SCR level can be seen as an indicator for vancomycin clearance and should be included in the PPK model development. However, among the population with ARC, due to the augmented permeability of lipid membrane, vancomycin can be transported into kidney at a much higher speed. The vancomycin mainly exists in kidney with 90% remaining unchanged. Plus, the clearance rate of vancomycin in kidney is much slower than that in plasma and renal tubules. All these factors lead to the loss of synchronicity between the blood vancomycin concentration and SCR level. Thus, SCR as a covariate was finally excluded from the final model in Model 2.

The results of our study suggest that for infant population whose GFR ≥ 86 ml/min/1.73 m^2^, only body weight data are sufficient for constructing vancomycin predictive models. Thus, taking blood sample is not compulsory for predicting vancomycin blood concentrations, which avoids unnecessary injury to vulnerable infants.

There are some limitations to this study. The first question should be addressed is why Model 1 and Model 2 did not show predictive improvement over Model 3 as we expected. Before model building process, we expected that models built on population sharing similar physiological condition can perform better when predicting the same population. However, the results seem to not support our hypothesis. We believe that this does not mean that our hypothesis should be denied. Our subgroups were extracted from a larger group; thus, the larger group contains all the information that the subgroups have. Thus, they possess same predictive ability at last. We will delve into this question in our future study by separately recruiting comparable amount of patients for Model 1, Model 2, and Model 3, rather than extract Models 1 and 2 population data from Model 3. The second limitation is that the normal renal function classification for infants is quite sophisticated. We choose the range of 30–86 ml/min/1.73 m^2^ to carry out our research because most of our patients are in the age range of 37–95 days. Our simplification to 30–86 ml/min/1.73 m^2^ may lead to model inaccuracy for certain infant age groups. The third limitation is that we used a one-compartment model rather than a two-compartment model, which was reported several times in previous studies and may better fit for the PK characteristics of vancomycin, to simplify the model building process. The simplification to one-compartment model may lead to deviation of clearance estimation. However, observations from the models show that the bias is acceptable. The fourth limitation is that the influence of infectious disease type on vancomycin pharmacokinetics was not evaluated. In previous studies, disease type was included as a significant covariate (20), but in this research, disease type was not studied. This may lead to some missing important variables that should be included in the final model.

To sum up, this study proposed a set of vancomycin PPK models based on renal function levels by using data from infants who were prescribed with vancomycin. Among infants with normal renal function level, WT and SCR were identified as significant covariates, while among infants with ARC level, WT was the sole significant covariate. Based on the results, for an infant whose eGFR ≥86 ml/min/1.73 m^2^, taking blood sample is not compulsory for predicting vancomycin blood concentration, which avoids unnecessary injury to vulnerable infants.

## Data Availability Statement

The raw data supporting the conclusions of this article will be made available by the authors, without undue reservation.

## Ethics Statement

The studies involving human participants were reviewed and approved by Ethics Committee of the Children's Hospital of Nanjing Medical University. Written informed consent from the participants' legal guardian/next of kin was not required to participate in this study in accordance with the national legislation and the institutional requirements. Written informed consent was not obtained from the minor(s)' legal guardian/next of kin for the publication of any potentially identifiable images or data included in this article.

## Author Contributions

D-YL, FC, and XinJ: conceptualization. D-YL, LL, and FC: methodology and investigation. D-YL and LL: software, validation, and formal analysis. G-ZL, Y-HH, H-LG, and XiaJ: resources and data curation. G-ZL, Y-HH, H-LG, XiaJ, and H-RD: data curation. D-YL: preparation of the original draft. D-YL, FC, and JX: reviewing and editing the manuscript. FC and XinJ: supervision and funding acquisition. JX: project administration. All authors have read and agreed to the published version of the manuscript.

## Funding

This research was funded by the Specially Appointed Medical Expert Project of the Jiangsu Commission of Health (2019) and the Medical Science and Technique Foundation of Nanjing Health Commission (YKK20132). This study was also funded by Jiangsu Research Hospital Association for Precision Medication (JY202021).

## Conflict of Interest

The authors declare that the research was conducted in the absence of any commercial or financial relationships that could be construed as a potential conflict of interest.

## Publisher's Note

All claims expressed in this article are solely those of the authors and do not necessarily represent those of their affiliated organizations, or those of the publisher, the editors and the reviewers. Any product that may be evaluated in this article, or claim that may be made by its manufacturer, is not guaranteed or endorsed by the publisher.
